# Chemical Sensors Based on Two-Dimensional (2D) Materials for Selective Detection of Ions and Molecules in Liquid

**DOI:** 10.3389/fchem.2019.00708

**Published:** 2019-11-15

**Authors:** Chung Won Lee, Jun Min Suh, Ho Won Jang

**Affiliations:** Department of Materials Science and Engineering, Research Institute of Advanced Materials, Seoul National University, Seoul, South Korea

**Keywords:** liquid, chemical sensor, biosensor, graphene, transition metal dichalcogenides, 2D materials

## Abstract

Up until now, two-dimensional (2D) materials have been researched vigorously for application to sensing ions and molecules in liquid due to their unique structural, chemical, and electronic properties. Features of 2D materials such as high surface area-to-volume ratios and various reaction sites are ideal characteristics for fabricating state-of-the-art high-performed chemical sensors. This review particularly focuses on the detection of pH, metal ions, and biomolecules in liquid media. The final goal of the ion/molecule sensors is a development of the electronic tongue or taste sensors that can be used in medical, food, biotechnology, and health applications. Herein, we introduce recent advances in the field of ion/molecule sensors in liquid media based on 2D materials, especially concentrating in graphene and MoS_2_, and will emphasize the opportunities and challenges of these unique sensing materials and devices.

## Introduction

A recent development in sensor technology and increasing demands in high-quality life have led to the era of the Internet of Things (IoT). The interconnected sensor devices and numerous data acquisition from various sources including the human body, indoor air, foods, infrastructures, and so on can provide real-time information of harmful substances or human body status with adequate actions to take upon those signals (Nowogrodzki, 2018). Among various fields of sensor technologies, the development of smart sensors mimicking human organs has attracted a tremendous amount of attention since electronically replicated human organs can examine various target substances with extremely lower detection limit and higher sensitivity, leading to early detection of the harmful agents. The human five senses include sight, hearing, smell, taste, and touch, and they respectively have a different approach to be electronically replicated in various research fields. The sight and hearing sensors have already been developed to a high level enough to be commercialized such as eye-tracking technologies and speech recognition in mobile phones and automobiles (Itti, 2015). The remaining smell, taste, and touch senses also have been intensively studied to develop sensors mimicking the human nose, tongue, and skin but still require further researches to be in real use as sight and hearing are.

Among the remaining three human senses, there has been significant progress in developing an electronic tongue (e-tongue) mimicking the human taste sense. Unlike electronic nose or skin aiming in various gaseous substances and external stimuli, respectively, e-tongue targets distinctive ions, or molecules (glucose, hydrogen ion, quinine, sodium ion, and monosodium glutamate) representing each of the five tastes (sweet, sour, bitter, salty, and umami). Although some companies have already commercialized equipment analyzing the above five taste substances, they have a relatively large scale to be adopted into the IoT platform, where small size, low power consumption, low price, and compatibility to existing circuits are essential requirements.

The two-dimensional (2D) materials are emerging candidates to be used in sensing devices and, further, can be applied to the IoT platform due to their promising physical and chemical properties. Their high surface area-to-volume ratio leads to high sensitivity to target substances. 2D material-based sensors can also be fabricated in a miniaturized size due to their flexibility, great mechanical strength, and optical transparency (Novoselov et al., 2005; Castellanos-Gomez et al., 2010; Gorbachev et al., 2011; Wang et al., 2012; Butler et al., 2013). For practical use of 2D material-based sensors, many requirements should be considered, but the selectivity issue should be the primary consideration. The approaches in the aspect of characteristics of sensing materials are the most effective way to improve ions or molecule selectivity since the selectivity highly depends on the nature of 2D materials and ion/molecule. 2D materials interact with target molecules/ions by two distinctive mechanisms: physisorption and chemisorption. Physisorption happens when the molecules/ions interact with the surface of 2D materials without any covalent bonding, and chemisorption refers to the covalent interactions between molecules/ions and the surface of 2D materials. When non-covalent interactions are preferred, it results in quick response and fast recovery. However, biomolecules such as DNA requires immobilizing processes on the surface, which makes covalent linking more preferable. The physisorption of molecules/ions onto 2D materials surface depends on the properties of both analyte and surface. For example, graphene has a honeycomb structure with a linkage of sp^2^ hybridized carbon atoms by a long-range π-conjugation. As a result, the non-covalent interaction between the surface of graphene and ions/molecules involving π bonds is important since the electronic characteristics of π systems can modify the structure or properties (Georgakilas et al., 2012). On the other hand, a representative transition metal dichalcogenide (TMD), MoS_2_, exhibits physisorption onto its basal planes due to the interactions between the ion/molecule and MoS_2_ via van der Waals force (Moses et al., 2009). Chemisorption can be induced by forming defects on the surface of the 2D materials. For example, graphene has point defects such as the absence of some sp^2^ carbon atoms or carbon atoms having sp^3^ hybridization. These defects induce different electronic structures from the original sp^2^ carbons resulting in an alteration of chemical reactions. These point defects increase the chemical reactivity of graphene. Furthermore, defects can be exploited to form nano-sized pores, permselective membranes (i.e., ion exchanger), or ultrasensitive sensors that can detect a sequence of DNA. Chemical functionalization of 2D materials can also enhance selectivity toward target substances.

**Figure 1:**
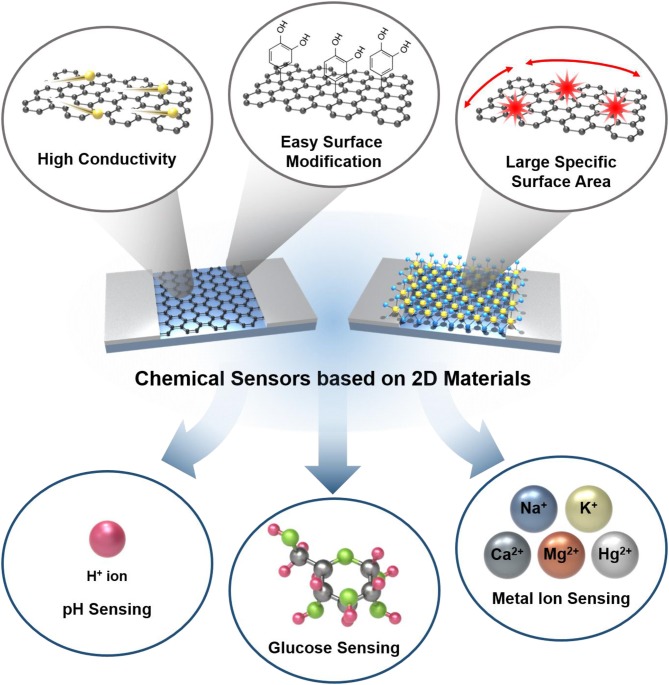
Schematic illustration of chemical sensors based on 2D materials with advantages of 2D materials and their application into ion/molecule sensing.

Herein, we summarized various studies and efforts on developing ion/molecule sensors based on 2D materials, especially focusing on graphene and MoS_2_; our scope is illustrated in Figure 1. Due to promising characteristics of 2D materials as above, their application to sensors has been conducted explosively in various approaches targeting numerous substances. Although there have been good reviews on 2D material-based sensors targeting gaseous molecules, only a few summarized those targeting ions and molecules existing in liquid media, which require much more complicated sensing mechanism and sensor structures. In this review, various 2D material-based sensors targeting ions and molecules including hydrogen ion, glucose, metal ions, organic molecules, and biomolecules are summarized with their sensor performances in the platforms of field effect transistors to help understand the current progress of the state-of-the-art ion/molecule sensors based on 2D materials.

## Hydrogen Ion Sensing

The most fundamental ion/molecule sensor is the hydrogen ion (H^+^) sensor (i.e., pH sensor). Hydrogen ion sensing is a vital measurement in many liquid chemical procedures such as industrial, manufacturing, pharmaceutical, and food production. The acidity of a substance may act as an indicator in many cases; food spoilage indication, monitoring clean/wastewater, chemical laboratory analysis, etc. pH meters commonly used in scientific laboratories are also called potentiometric pH meters. The basic fundamental of pH meters is measuring the electrical potential difference and monitoring the hydrogen ion concentration of a typical solution. This method is very precise in sensing the concentration of hydrogen ions, but there is a critical disadvantage that the device is not portable due to its size and the necessity of external power source. Explained above, IoT devices must be miniaturized and portable for everyday use. 2D materials can be used as pH sensors because of their high sensitivity from their chemical and physical properties. Additionally, hydrogen ion sensors based on 2D materials can be minimized in size, making them emerging candidates of hydrogen ion sensors.

### Graphene

Graphene can electrically detect chemical species due to its single-layer thickness and high carrier mobility (Ohno et al., 2009; Cheng et al., 2010). Since graphene has a unique semi-metallic characteristic with a zero bandgap (Berger et al., 2006), graphene field-effect transistors (GFETs) reveal bipolar features in terms of conduction properties. GFETs exhibit distinct transfer characteristics in source–drain current vs. gate voltage (*I*–*V*_g_), which has a conductance minimum point called Dirac point (*V*_Dir_) or charge neutrality point (*V*_CNP_). In the case of *V*_g_ having a lower value than *V*_CNP_, the majority of charge carriers are holes and increasing *V*_g_ leads to the decline of channel current and, reversely, the majority charge carriers become electrons, which increases in quantity with increasing *V*_g_. Consequently, GFETs have the advantage of noticing the shift of charge neutrality point by adsorption or desorption of electrons or charged ions. Sensitivity can be estimated by evaluating the degree of horizontal shift of the charge neutrality point. In other words, as chemical alteration such as ion concentration or pH increase or decrease results in the surface of GFET, the *V*_CNP_ will change (Kwon et al., 2015).

**Figure 2:**
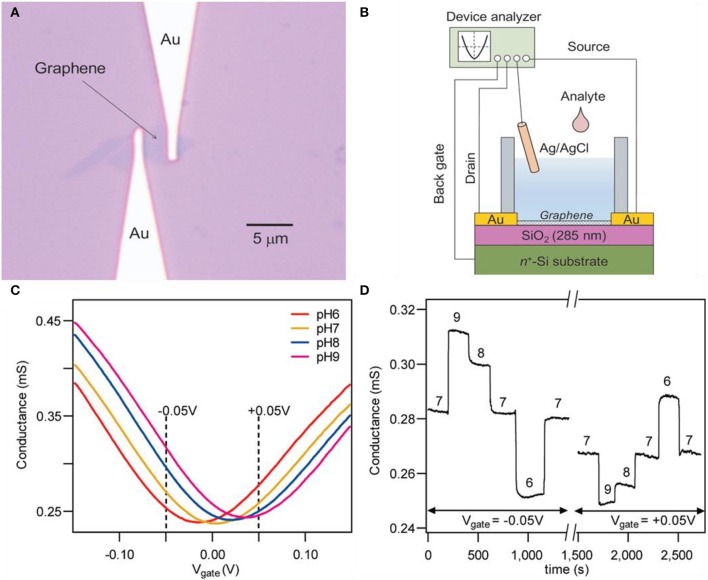
Pristine graphene pH sensors. **(A)** Optical microscope image of a graphene field effect transistor. **(B)** Experimental setup scheme illustration of a graphene field effect transistor. Figures **(A,B)** were reproduced from Ohno et al. (2009) with permission from the American Chemical Society. **(C)** Typical transfer curves and **(D)** time–current curves of a graphene field effect transistor. Figures **(C,D)** were reproduced from Cheng et al. (2010) with permission from the American Chemical Society.

Ohno et al. successfully demonstrated the transport behaviors of GFETs in pH solutions and protein molecules (Ohno et al., 2009). The prepared graphene was a single-layered crystal prepared by mechanically exfoliating natural graphite. Figure 2A indicates the optical microscope image of exfoliated graphene contacted with gold electrodes and Figure 2B depicts the overall setup of the experiment. The input electrolytes (pH solutions) into the well were made with mixing various types of buffer solutions for each pH value. The relationship between transfer characteristics and conductance of GFET on pH was revealed. By the shift of charge neutrality point toward the positive direction as the increment of pH, it could be assumed that the negative charge needed to reach the neutral point (minimum value) increases as pH rises since holes are the main charge carrier. Cheng et al. reduced the noise level and improved the signal-to-noise ratio by removing the oxide by suspending graphene in aqueous solutions for *in situ* etching (Cheng et al., 2010). After mechanical exfoliation of graphite, single-layer graphene was transferred to the silicon substrate. An etching process was progressed, preventing graphene from air exposure because any drying steps will generate stress and eventually deform the graphene. The deformation of the graphene will alter the electrical properties of it and will consequently cause graphene to malfunction. Buffed hydrofluoric acid (HF) was used for etching and the conductance of the device drastically declined after injection. After the drastic decrease of conductivity, it was stabilized in 50 to 100 s, designating the termination of the etching process. As shown in Figure 2C, transfer curves were depicted in the span of pH 6 to 9. These pH solutions are prepared with a combination of potassium chloride and phosphate solution. Charge neutrality points are shifted to the right as pH increases in Figure 2C. Conductivity value increases as pH increases in negative *V*_g_. But tendency reverses if the *V*_g_ has a positive value as shown in Figure 2D. This result can be confirmed in the graph of Figure 2C. In *V*_g_ = −0.05 V, conductivity is higher in higher pH, and in *V*_g_ = 0.05 V, conductivity is lower in higher pH. This is due to the ambipolar characteristics of graphene; mentioned above, in the case of *V*_g_ < *V*_CNP_, the main charge carrier are holes and higher pH will result in higher conductivity because OH^−^ ions induce holes to the surface of graphene. Reversely, in the case of *V*_g_ > *V*_CNP_, the main charge carriers are electrons and lower pH will result in higher conductivity because hydrogen ions induce electrons to the surface of graphene. Reversibility was confirmed by repeatedly injecting pH solutions in sequence. Zhu et al. designed a GFET without an external gate source (elimination of reference rod) by embedding a solid gate, suggesting the possibility of a practical analytic device using graphene (Zhu et al., 2015). A typical thin layer composed of HfO_2_ with a high dielectric constant (κ) is used as a gate dielectric layer. The HfO_2_ layer has higher specific capacitance compared to conventional SiO_2_ solid-gated sensors, leading to higher transconductance and operation at lower *V*_g_. Also, the HfO_2_ layer prevents the gate electrode to be exposed from the liquid, eliminating any errors (e.g., bulk motion of sample solution). This device does not require any separate components such as external reference rods, which simplifies the fabrication process. Using a low *V*_g_ (1.5 V), the device can measure pH in a span of 5.3 to 9.3 with a sensitivity of ~57 mV/pH.

Graphene oxide (GO) and reduced graphene oxide (RGO) are also candidates for pH sensing. GO is a 2D material that is originated from natural graphene by chemical conversion (Park and Ruoff, 2009). GO has various advantages such as being stable into single-layered sheets in water and being functionalized with ease. Also, GO is differentiated to graphene by its semiconducting features from the oxidation of graphene, leading to bandgap increase (Cai et al., 2008). There are various methods to synthesize GO such as the Staudenmaier (1898) and Hummers method (Hummers Jr and Offeman, 1958). These methods all include the oxidation process of graphite. To oxidize graphite, Staudenmaier mixed potassium chlorate (KClO_3_) with nitric acid (HNO_3_). On the other hand, Hummers treated graphite with a mixture of potassium permanganate (KMnO_4_) and sulfuric acid (H_2_SO_4_) (Zhu et al., 2010). Due to the polar oxygen functional groups of GO, exfoliation in many solvents is possible and dispersion by simple stirring or sonication is available with ease (Zhu et al., 2010). RGO has similar properties with graphene since the reduction process on GO involves removing oxygen functional groups. In other words, RGO possesses both advantages of pure graphene and GO: high conductivity and existence of chemically active defect sites (Robinson et al., 2008). There are a variety of methods to reduce GO to RGO; exposing GO to reducing chemicals such as hydrazine or to alkalis, hydrogen plasma treatment, thermal annealing, and photocatalytic reduction (Stankovich et al., 2006; Gómez-Navarro et al., 2007; Jung et al., 2008; Wang et al., 2008; Williams et al., 2008). GO and RGO are typically p-type materials, so when these materials are exposed to oxidizing gases (e.g., NO, NO_2_), the resistance of both materials declines (Choi et al., 2015).

**Figure 3:**
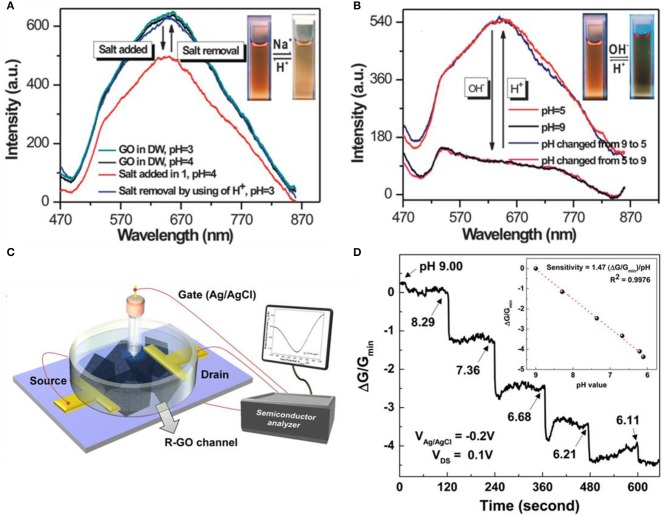
Graphene oxide (GO)- and reduced graphene oxide (RGO)-based pH sensors. **(A)** Reversible vis-NIR fluorescence change of acidic vs. salt states. **(B)** Reversible vis-NIR fluorescence change of pH. Figures **(A,B)** were reproduced from Chen and Yan (2011) with permission from the Royal Society of Chemistry. **(C)** Schematic diagram of an RGO field effect transistor. **(D)** Typical sensing curves along pH alter RGO. Figures **(C,D)** were reproduced from Sohn et al. (2013) with permission from Elsevier.

The most distinct feature of GO compared to pristine graphene and RGO is that, GO is electrically insulating (Fan et al., 2008) and a bandgap exists (Boukhvalov and Katsnelson, 2008; Eda et al., 2010). Due to these characteristics, rather gathering data with transfer curves or current vs. time curves, GO-based pH sensors are focused on luminescence features taking advantage of the presence of bandgap. Chen et al. revealed the reversibility of vis-NIR fluorescence under different conditions of acidic and salt (Na^+^) conditions or pH (Figures 3A,B; Chen and Yan, 2011). The protonation and deprotonation of the carboxylate functional group from GO lead to activation and deactivation of luminescence. When the state of the carboxyl functional group is –COOH, it becomes luminescent and if the state becomes –COO^−^, it alters to non-luminescent. Adding OH^−^ to –COOH will induce it to become –COO^−^, and on the contrary, applying H^+^ to –COO^−^ will change it to –COOH. To summarize, adding salt or –OH^−^ leads luminescence intensity to decrease. On the contrary, removing salt or applying H^+^ leads to luminescence intensity to increase. As depicted in Figures 3A,B, both reactions are reversible and eventually find their initial states. Bai et al. formed a composite hydrogel with GO and poly(vinyl alcohol) (PVA) for selective drug release at a particular pH (Bai et al., 2010). Various content ratio of GO/PVA mixtures was produced to find the ideal proportion. Theoretically, one PVA chain can be linked with two or more GO sheets, which can form cross-linking sites. If PVA content exceeds the ratio of 1:2 (= PVA: GO), the majority of PVA chains become adsorbed on both faces of a single-layer GO, which leads to weakening the cross-linking effect of PVA chains. The ideal ratio was 1:10 (= PVA: GO) for releasing drugs at physiological pH. The drug leakage ability of the GO/PVA composite was evaluated by loading vitamin B_12_ to the hydrogel and testing in various pH conditions. Rather than acidic conditions, releasing in neutral conditions have higher efficiency. Due to the pH-sensitive properties of GO, drugs can be released to preferred physiological pH.

Possessing both merits of pristine graphene and GO, RGO is also a candidate for pH sensors. The surface and edges on the RGO nanosheets contain oxygen functional groups that will enhance the sensing response of pH (Sohn et al., 2013). Sohn et al. reduced manufactured GO nanosheets by the Hummers method. After the exfoliation process of GO by sonication, it was spin-coated to the SiO_2_ substrate and a channel area was formed. After the drying process, the GO was reduced with hydrazine monohydrate to produce a conductive RGO channel. After annealing at 200°C for 2 h in Ar atmosphere, for additional functional groups, the RGO channel was immersed in dimethylformamide (DMF) containing 1-pyrenebuthanoic acid succinimidyl ester at room temperature (RT) for 2 h. Buffer solutions were used by mixing 0.1 M phosphate buffer and 0.1 M NaCl solution. Figure 3C illustrates the overall setup of the RGO channel device. The sensing curves of the RGO channel are depicted in Figure 3D, having lower sensitivity toward lower pH. Pristine graphene also has an identical tendency of RGO in terms of sensing pH. Their sensing mechanism is equal in the point of view of H^+^ binding to the surface, eventually inducing the surface charge density to change on the gate. Precisely, the processes are different. In the case of pristine graphene, when exposed to acid electrolytes, hydronium ions (H_3_O^+^) attach to the inner Helmholtz plane (interface formed when an electrolyte and electronic conductor meets), inducing the graphene to become electron doped. On the other hand, when exposed to alkali electrolyte, hydroxyl (OH^−^) attaches to the inner Helmholtz plane and the graphene becomes hole doped (Lei et al., 2011). Both H_3_O^+^ and OH^−^ ions are non-faradic (capacitive), so these charges are impermeable across the graphene/electrolyte surface (Ang et al., 2008). As the H_3_O^+^ ions or OH^−^ ions are distributed to the Helmholtz inner plane, OH^−^ ions are more orderly arranged in the inner plane than H_3_O^+^ ions. Therefore, the conductivity value has a proportional relationship with pH (Ang et al., 2008). RGO also has functional groups such as –OH, –COOH on the surface similar to GO and high conductivity, which is the keystone of the sensing mechanism. These groups have interactions with H^+^ ions in the electrolyte. In lower pH buffer solutions, more H^+^ combines with O^−^ and COO^−^, consequently leading the positive charge to incline on the surface of RGO. This induces the increment of electrons in the channel, which leads the *V*_CNP_ to move in the negative direction (Sohn et al., 2013).

### TMDs and Other 2D Materials

**Figure 4:**
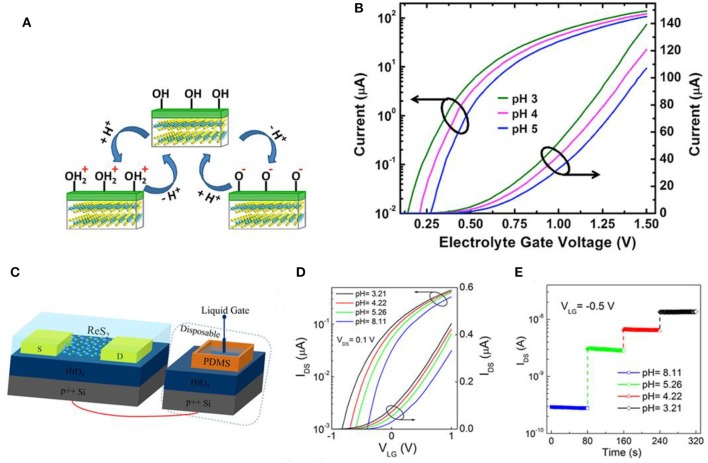
MoS_2_ FET and ReS_2_ FET pH sensors. **(A)** Sensing mechanism of MoS_2_ pH sensors. At low pH, ${\text{OH}}_{2}^{+}$ forms by protonation of OH on the dielectric surface, consequently forming a positive surface charge on the dielectric. At high pH, O^−^ forms by deprotonation of OH on the dielectric surface, consequently forming a negative surface charge on the dielectric. **(B)** Drain current vs. electrolyte gate voltage in various pH values for an n-type MoS_2_ FET. Figures **(A,B)** were reproduced from Sarkar et al. (2014) with permission from the American Chemical Society. **(C)** Illustration of ReS_2_ FET for pH sensing. **(D)** Transfer curves (*I*_DS_-*V*_LG_) at various pH values. **(E)** Response curves at different pH with *V*_DS_ = 0.1 V and *V*_LG_ = −0.5 V at RT. Figures **(C–E)** were reproduced from Liao et al. (2018) with permission from the American Chemical Society.

The most representative TMD is molybdenum disulfide (MoS_2_), composed of Mo (transition metal) and S (chalcogenide atom), which are covalently bonded. These 2D stacked layers are arranged in a row and they are held together by weak van der Waals force. This weak force makes exfoliation rather easier, and single-layered film extraction is possible (Sarkar et al., 2014). The mechanism of TMD-based devices detecting pH alteration is similar to GFET. Sarkar et al. measured the current change as pH alters and proposed MoS_2_ as a candidate for a typical H^+^ ion sensor (Sarkar et al., 2014). As shown in Figure 4A, the main sensing mechanism is the protonation and deprotonation of the OH groups on the surface of the gate dielectric by the pH of the electrolyte. In the case of low pH, the surface will be protonated (OH + H^+^ = ${\text{OH}}_{2}^{+}$), generating positive charges while deprotonation occurs on the surface (OH – H^+^ = O^−^) in high pH resulting in negative charges. In Figure 4B, electrolyte gate voltage vs. drain current curves is depicted for various pH values. At a fixed applied bias, the decline of pH value will lead to the incline of current (i.e., dielectric surface having a higher positive charge inducing to lower the threshold voltage of FET) and vice versa. This threshold voltage shift can be explained by surface charge alter due to pH change. Liao et al. used layered rhenium disulfide (ReS_2_) for low-frequency noise field effect transistors for pH sensing (Liao et al., 2018). The ReS_2_ flakes were obtained by mechanically exfoliating commercial bulk ReS_2_ crystals. Illustrated in Figure 4C, the diagram reveals the overall structure of ReS_2_. An Ag/AgCl electrode was used for the liquid gate and a thin HfO_2_ gate dielectric was used to hold phosphate buffered saline (PBS) solution, respectively. The thickness of HfO_2_ was 20 nm, which was deposited by atomic layer deposition (ALD) on highly doped Si. Figure 4D indicates the transfer curves of the pH sensor, which shifts in a positive direction as the pH value increases. Higher pH value in the solution induces a more negative charge on the surface of the HfO_2_ gate. Figure 4E shows response curves (*I*_DS_ vs. time) on various pH values. The source–drain and the gate voltage are fixed to *V*_DS_ = 0.1 V and *V*_LG_ = −0.5 V, and current changes drastically and stabilizes quickly in each pH value. Shadman et al. used MoS_2_ and WSe_2_ as a double gate field effect transistor for pH sensing (Shadman et al., 2016). The sensitivity is influenced by the thickness of back and top oxide. The thicker the back oxide and the thinner the top oxide, the higher the sensitivity of the device. Nasir et al. investigated the alteration of inherent oxidative peaks in various pH values of different TMDs (Zafir Mohamad Nasir et al., 2015). Using potential–pH diagrams (Pourbaix diagram), this group found MoS_2_ and MoSe_2_ becoming oxidized from +4 to +6 states at pH range ~8. Also, until pH 11, the peak position remains almost stable, but the shift of the peak is considerable. The peak of WS_2_ was almost similar to the pH range of 2–12. The tungsten-based dichalcogenides possessed better stability than molybdenum-based dichalcogenides.

## Glucose Sensing

Glucose is a vital component in sitology, physiology, and biotechnology. Glucose is also the most basic and important energy source of human daily life. If glucose concentration decreases in the human body, people may go under shock. On the other hand, an excessive amount of glucose in the blood results in diabetes, which is an enormous problem these days. In both cases, glucose monitoring is crucial, which many researchers are focusing on fast, reliable glucose sensors for healthcare monitoring. In the 1960s, Clark and Updike first reported the enzyme electrode (Clark and Lyons, 1962) and biosensor (Updike and Hicks, 1967), respectively. After these studies, glucose sensors based on glucose oxidase (GOx) have been researched.

### Graphene

**Figure 5:**
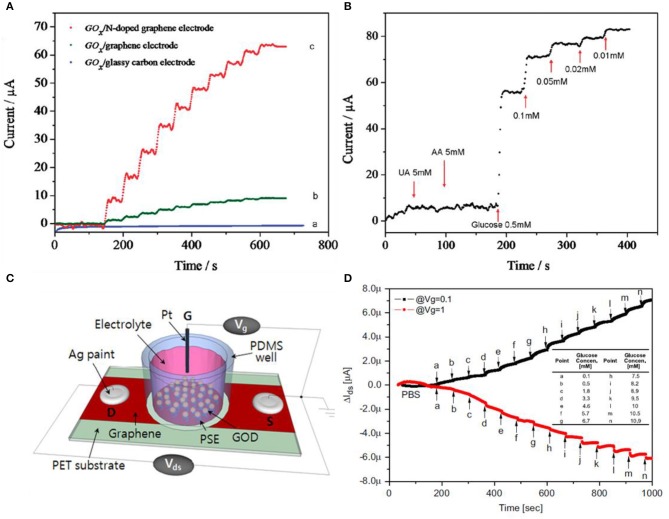
Glucose sensors using graphene-based materials. **(A)** Glucose responses to various graphene-based materials. **(B)** N-doped graphene having selectivity to glucose than uric acid and ascorbic acid. Figures **(A,B)** were reproduced from Wang et al. (2010) with permission from the American Chemical Society. **(C)** Schematic illustrations of the CVD graphene FET sensor. **(D)** Response curves of glucose in different gate voltages. Source–drain voltages are fixed to *V*_DS_ = −0.2 V. Figures **(C,D)** were reproduced from Kwak et al. (2012) with permission from Elsevier.

Graphene-based materials are promising candidates for glucose sensing, but rather than using pristine graphene, decorating metal nanoparticles or forming a composite form using chitosan is much more favorable for selectivity. Using functionalized GO or RGO is also an alternative. Wang et al. used nitrogen-doped graphene for selective detection toward glucose by treating graphene with nitrogen plasma (Wang et al., 2010). The nitrogen percentage was controlled by plasma exposure time, consisting of 0.11–1.35%. N-doped graphene has the advantage of having high electrocatalytic activity for H_2_O_2_ reduction and fast, direct electron transfer to GOx. GOx can catalyze the oxidation process of glucose to gluconic acid and H_2_O_2_ with oxygen. In other words, glucose sensing is equivalent to the detection of H_2_O_2_ during the enzymatic catalytic activity. As shown in Figure 5A, three different electrodes were compared by the performance of the catalytic effect. The measurements on each type were conducted at −0.15 V in 0.1 M physiological buffer solution with a consecutive addition of 0.1 M glucose. Also, in Figure 5B, selectivity of sensors was investigated by adding uric acid (5 mM) and ascorbic acid (5 mM). After confirming no response to uric and ascorbic acids, glucose was added from high concentration to low, obtaining linear responses for each concentration. Kwak et al. used chemical vapor deposition (CVD)-grown graphene for glucose sensing (Kwak et al., 2012). The interesting point is that for a flexible device, this group choose polyethylene terephthalate (PET) as a substrate. As illustrated in Figure 5C, conductive silver paste was covered on both ends of the graphene thin film. A well-type polydimethylsiloxane (PDMS) was attached on top of the graphene to confine the solutions from leaking out. Then, graphene was then surface modified with GOx for catalytic effects when sensing glucose. The gate voltage is applied from the top gate electrode, which induces the accumulation of ions at the boundary between graphene and solution. Graphene also has ambipolar features, explained previously on the pH sensor section, that can be operated both in n- and p-type regions. If the charge neutrality point (*V*_CNP_) is higher than the gate voltage (*V*_g_), graphene is in the circumstance of p-type, and in contrast to this, graphene behaves as an n-type material. Figure 5D indicates that *V*_CNP_ is in between 0.1 V and 1.0 V. In other words, in the case of *V*_g_ = 0.1 V, it is p-type, and at *V*_g_ = 1.0 V, it is n-type. In both cases, *V*_DS_ = −0.2 V and an identical amount of glucose was injected with higher concentration each step. Kang et al., Shan et al., and Wu et al. all manufactured the sensor by fabricating a nanocomposite with GOx (enzyme)–graphene–chitosan. Wu et al. additionally added platinum nanoparticles for higher sensitivity while Shan et al. added Au nanoparticles. Kang et al. dispersed graphene in chitosan solution using ultrasonication. Then, GOx was coated on the graphene/chitosan film (Kang et al., 2009). Wu et al. added Pt nanoparticles to enhance the response of glucose compared to the un-decorated sample (Wu et al., 2009). Shan et al. also had a property increment of glucose sensing using the decoration of Au nanoparticles (Shan et al., 2010).

Using graphene as well as using GO and RGO are alternatives for glucose sensing. In particular, carboxyl-modified GO possesses the ability to intrinsically catalyze the peroxidase reaction (Song et al., 2010). Song et al. noticed the peroxidase process by checking GO-COOH turning into blue when catalytic activity happens. The optimal pH was 4.0, the temperature was 35°C, and 150 mM of H_2_O_2_ concentration. Also, this sensor has selectivity to glucose; fructose, lactose, and maltose have no response. Wang et al. performed a typical RGO glucose sensor by electrochemically reducing GO (Wang et al., 2009). GO has the advantage of having oxygen functional groups such as –OH, –COOH, and epoxides leading to hydrophilic features but GO is not compatible with organic polymers. GO is chemically modified with GOx using a polymer called *N*-succinimidyl acrylate. Luo et al. designed a non-enzymatic glucose sensor based on copper nanoparticle-decorated graphene sheet (Luo et al., 2012). The introduced former researches all involve GOx, which is an enzyme, but this paper implies the probability of non-enzymatic glucose sensors. The copper nanoparticles were modified on graphene by electrodeposition. Copper electrodeposition was optimized by modulating the depositing voltage (deposition time was fixed to 400 s) by comparing 0.2 V−0.55 V. 0.5 V was chosen as the optimal value, and response curves were obtained having repetitive, sensitive, stable, and selective results.

### TMDs and Other 2D Materials

**Figure 6:**
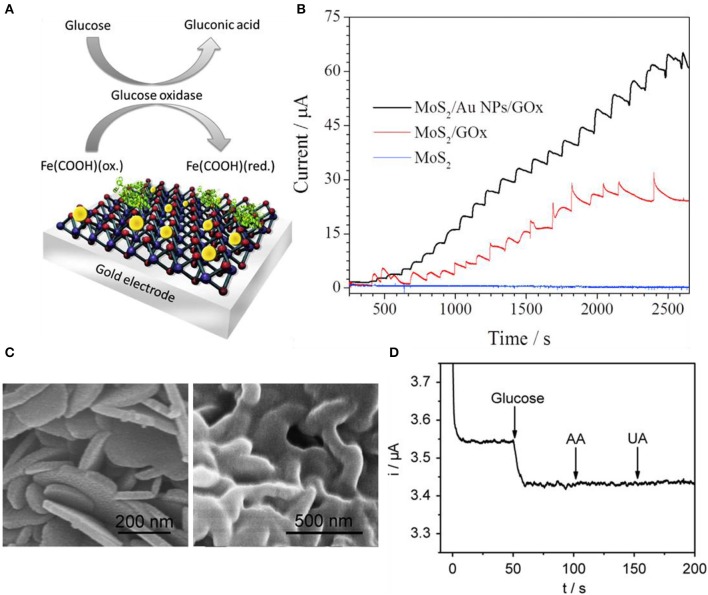
Glucose sensors using transition metal dichalcogenides. **(A)** Illustration for basic glucose sensor using Au nanoparticle decorated MoS_2_. **(B)** Response comparison between pristine MoS_2_, MoS_2_/GOx, and MoS_2_/Au NP/GOx electrodes. Figures **(A,B)** were reproduced from Parlak et al. (2017) with permission from Elsevier. **(C)** Scanning electron microscope images of pristine SnS_2_ (left) and GOx/MWCNTs–SnS_2_ (right). **(D)** Response curves of glucose having selectivity at GOx/MWCNTs–SnS_2_. Figures **(C,D)** were reproduced from Li et al. (2013) with permission from Elsevier.

As graphene, TMDs can also detect glucose with decorated nanoparticles. In terms of electrochemical viewpoint, nanoparticle decoration on the electrode surface not only helps electrocatalytic reactions but also presents electrochemical properties. These properties are faradic to capacitive current ratios, electron mobility, current density, and mass transport (Huang et al., 2008; Peng et al., 2009; Zeng et al., 2011). Due to these advantages, nanoparticle decoration or deposition is applied to many cases (Jeon et al., 2017). In general, there are three methods to decorate nanoparticles on surfaces including electrochemical etching, nanoband electrode fabrication, and electrodeposition of metal nanoparticles. Electrodeposition is relatively cost-effective compared to the other two methods, so the electrodeposition method is widely used. Still, electrodeposition has multiple signs of progress and may need special chemical treatments and particular conditions. Parlak et al. used Au nanoparticles to enhance selectivity toward glucose using a mixture of MoS_2_ nanosheets and Au nanoparticles by sonication (Parlak et al., 2017). Figure 6A indicates the schematic diagram of the Au nanoparticle-decorated MoS_2_ and proposed sensing mechanism. MoS_2_ nanosheets were dispersed in a 10 mM phosphate buffer solution and were treated with ultrasonication. In Figure 6B, amperometric responses were compared between MoS_2_/GOx and MoS_2_/Au nanoparticles/GOx electrodes in 0.1 M phosphate buffer solution and the applied voltage was 0.35 V. MoS_2_-modified nanosheet electrodes had no electrocatalytic response to any concentration of glucose while MoS_2_/GOx and MoS_2_/Au nanoparticles/GOx electrodes exhibited a response. Au nanoparticle-doped cases have better sensitivity to glucose. Su et al. also utilized Au nanoparticles for glucose sensing enhancement. The difference between Parlak et al. and Su et al. is that Parlak et al. dispersed Au nanoparticles with ultrasonication while Su et al. synthesized Au nanoparticles onto MoS_2_ by the hydrothermal method (Su et al., 2014). Sensitivity and selectivity were investigated by injecting various solutions such as uric acid, dopamine, NaCl, KCl, and glucose. Only glucose had a response to the device. Huang et al. sought electrocatalytic effects from Ni nanoparticles rather than Au nanoparticles (Huang et al., 2014). Ni nanoparticles were reduced on MoS_2_ nanosheets by a solution process. Amperometric response with glucose, dopamine, ascorbic acid, and uric acid was investigated for response sensitivity and selectivity. Lin et al. decorated copper nano-flowers to MoS_2_ nanosheets. The copper was decorated using the electrodeposition process. H_2_O_2_ was selectively sensed while ascorbic acid, dopamine, fructose, lactose, and uric acid had no response (Lin et al., 2016). Cai et al. formed a MoS_2_-PtAg nanohybrid for colorimetric detection of glucose (Cai et al., 2016). Bimetallic nanoparticle (BNP) supported on MoS_2_ is much more uncommon than single-metal nanoparticle supports due to the complexity of forming composites. Bimetallic nanoparticle decorations perform great catalytic effects due to their synergistic effect between two different metals. Recently, many groups are finding a simple method to synthesize bimetallic nanoparticle composites on MoS_2_ or other 2D materials (Zhong et al., 2012; Sun et al., 2015; Su et al., 2016) but MoS_2_-BNP hybrids with high quality and various heterostructures forming remain a challenge. Cai et al. prepared MoS_2_ with liquid exfoliation and functionalization by polyallylamine hydrochloride (PAH). After forming MoS_2_-PAH nanosheets, metal nanoparticles were hydrothermally reduced to the nanosheets. Two different metal precursors were prepared for Pt and Ag, respectively. Experimental results were confirmed by color change (blue) by each concentration of H_2_O_2_.

Not only MoS_2_ but also other TMDs and 2D materials are used as glucose sensors. SnS_2_ is an n-type semiconductor having a bandgap of 2.18–2.44 eV. They are resistive to acids and have good stability in air, which has the capability of photocatalysts. Li et al. synthesized nanoflake SnS_2_ shown in Figure 6C (Li et al., 2013). The left SEM image is pristine SnS_2_ nanoflakes and the right is GOx/MWCNTs (multiwall carbon nanotube)-SnS_2_. MWCNTs were initially mixed into nitric acid, and then after the centrifuging process, it was neutralized. These carbon nanotubes were then dispersed with yellow nanoflake SnS_2_ in water and vigorously mixed using ultrasonication. Then, GOx was dropped to form composites with MWCNT–SnS_2_. After fabrication, the reproducibility and stability of the MWCNT–SnS_2_ sensor are demonstrated in Figure 6D. Amperometric responses were shown and had a response to only glucose while having no response to ascorbic acid and uric acid. All solutions were 0.1 mM in pH 6.0 phosphate buffer solution applying −0.43 V.

## Metal Ion Sensing

Due to population growth, increase in industrial and agricultural activities led to pollution in the environment and ecosystem. Recently, pollutant portable sensors have been studied in diverse ways to detect and eliminate the pollutants. The most representative pollutant from human activity is heavy metals leaking to the environment and threatening the ecosystem by inducing various diseases (Ali, 2012; Sholl and Lively, 2016). 2D materials can detect various metal ions by electrical interaction and adsorption opening a new era of water purifying (Aliprandi et al., 2017). 2D materials are single-layered and have two surfaces for adsorption having the advantage of the extremely high surface area-to-volume ratio, which leads to fast response, quick recovery rate, and multiple usages. In particular, GO can remove metal ions due to its hydrophilic feature and functional groups including oxygen atoms that can bind metal ions to the surface (Zhao et al., 2011; Sitko et al., 2013). TMDs, on the other hand, can potentially absorb certain heavy metals due to their numerous intrinsic chalcogen atoms. Especially, 2D material nanosheets integrated into FETs have recently been researched and their high sensitivity toward heavy metals was revealed. The basic sensing mechanism is the change of electrical parameters of a FET including 2D material nanosheets when the device adsorbs the typical heavy metal. These electrical parameters include field effect mobility, on current/off current ratio, and threshold voltage. It is well known that semiconducting 2D material nanosheets have high charge carrier mobility and surface energy that cause high sensitivity toward various materials. Other chemical sensors that do not involve FETs that use optical methods have the disadvantage of complicated processes or the need for chemical agents and long detection time. However, FETs have an extremely short detection time (real-time measurement available) by monitoring the resistance/conductance increase/decrease or the shift of charge neutrality point by the adsorption of the target substance. These devices can be miniaturized and can be developed as portable sensors supported on flexible substrates.

### Graphene

**Figure 7:**
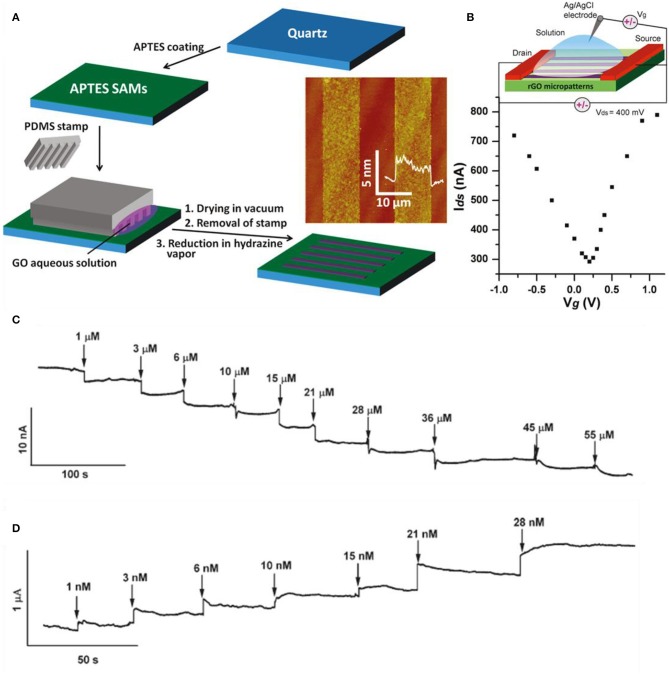
Graphene-based FET metal ion sensors. **(A)** Fabrication process of RGO thin films on 3-aminopropyltriethoxysilane (APTES)-modified quartz. **(B)** *I*_DS_-*V*_g_ curve at 0.1 M phosphate buffer solution of RGO FET. Inset: Schematic diagram of overall RGO FET. **(C)** Real-time sensing curves with various concentrations of Ca^2+^ ions. **(D)** Real-time sensing curves with various concentrations of Hg^2+^ ions. Figures **(A–D)** were reproduced from Sudibya et al. (2011) with permission from the American Chemical Society.

Graphene may be used as the sensing material in the channel positioning in between the drain and source electrodes. The gate potential can be applied from the top or bottom, in which the former is applied to the electrical double layer in the electrode and the latter is applied through a typical thin SiO_2_ substrate. As the molecule/ion becomes absorbed by graphene, the local charge characteristics change and conductance is altered. Sudibya et al. fabricated reduced GO for the reasons of cost-effectiveness, solution process availability, and scalable production (Sudibya et al., 2011). Micropatterning RGO film and functionalizing it with protein give the possibility to detect diverse metal ions with high sensitivity in real time. In Figure 7A, the fabrication process is illustrated as well as the micropatterning process of RGO on 3-aminopropyltriethoxysilane (APTES)-modified quartz. Then, the RGO thin films were functionalized with calmodulin (CaM). Figure 7B shows the ambipolar features of RGO measured in 0.1 M phosphate buffer saline and the inset is the scheme of overall solution-gated RGO-FET. Various metal ions were detected such as Ca^2+^, Mg^2+^, Hg^2+^, and so on. As shown in Figure 7C, Ca^2+^ ions caused conductance decline in the functionalized RGO-FET in a concentration-dependent behavior. By the difference of gate voltage, the RGO-FET has both characteristics of n-type and p-type, which is noticed by the conductance incline or decline by the injection of metal ions. The FET also bound with Mg^2+^, but having a lower affinity. Both ions (Ca^2+^, Mg^2+^) can be bonded onto RGO by surface modification. However, K^+^ and Na^+^ had no response, indicating that the functional group (CaM) has no selectivity to K^+^ and Na^+^. Also, without functionalization (CaM), the RGO-FET had no response to Ca^2+^ and Mg^2+^, meaning CaM is crucial for selective detection. Also, other heavy metal detection experiments were progressed by functionalizing RGO with metallothionein type II protein (MT-II), which binds with Zn, Cu, Se, or Cd, and Hg selectively. As shown in Figure 7D, sensing curves were depicted by the addition of Hg^2+^ at a detection limit of 1 nM. Comparing the detection of Ca^2+^ and Hg^2+^, mercury has much higher sensitivity than calcium, which indicates the fact that mercury has better binding affinity to MT than calcium to CaM. MT-II is negatively charged in pH 7.0, and metal binding brings MT-II closer to the RGO surface and increases the field effect from highly negative charged MT-II to RGO. As shown in Figures 7C,D, the RGO sensors are very stable in each step, having consistent conductivity. Sofue et al. detected sodium ions with a typical graphene FET (Sofue et al., 2011). Sodium ions in the electrolytes influence the electrical potential of the graphene channel. The graphene was mechanically exfoliated with adhesive tape. After exfoliating graphene on Si substrate, electrodes were formed for source and drain and a rubber frame was adhered on top of the graphene to block the leakage of electrolyte. Tris(hydroxymethyl)aminomethane and HCl (Tris–HCl) buffer solution was used as the solution. Various concentrations of NaCl were added to the buffer solution to supply Na ions. The source drain voltage was set at 0.1 V and the NaCl concentration range was 0 to 6.0 mM. Sodium ions adsorb/desorb the hydroxyl groups from the graphene surface. As Na^+^ ions increase on the graphene surface by the addition of NaCl solution, the positive charge accumulates on the graphene channel. On the other hand, when Na^+^ ion concentration is low, these ions adsorb to the hydroxyl group. Consequently, the charge neutrality point shifts toward the negative direction as the concentration increases. Transfer curves in diverse Na^+^ concentrations were plotted and curve shifting happened. Also, the current–time graph was plotted and the drain current decreased as Na ion concentration increased. Wen et al. detected lead ions using Au nanoparticles and DNAzyme functionalization onto graphene (Wen et al., 2013). Au nanoparticles were decorated initially on graphene due to DNAzyme molecule immobilization. DNAzymes have the advantage of low cost, high stability, and selectivity compared to protein and RNA enzymes. Many DNAzyme molecules adhere to a single Au nanoparticle. Graphene transfer curves shift positively in a large order when decorated with Au nanoparticles (reduced from HAuCl_4_) due to p-type doping effect. DNA molecule induces n-doping effects to graphene and curves shift to the negative direction. Consequently, the DNAzyme/Au nanoparticles/graphene curve is in the intermediate of graphene and Au nanoparticles/graphene curves. When lead ions become adjacent to the device, the transfer curves shift to the right side again and the shifting is proportional to the concentration of lead.

### TMDs and Other 2D Materials

**Figure 8:**
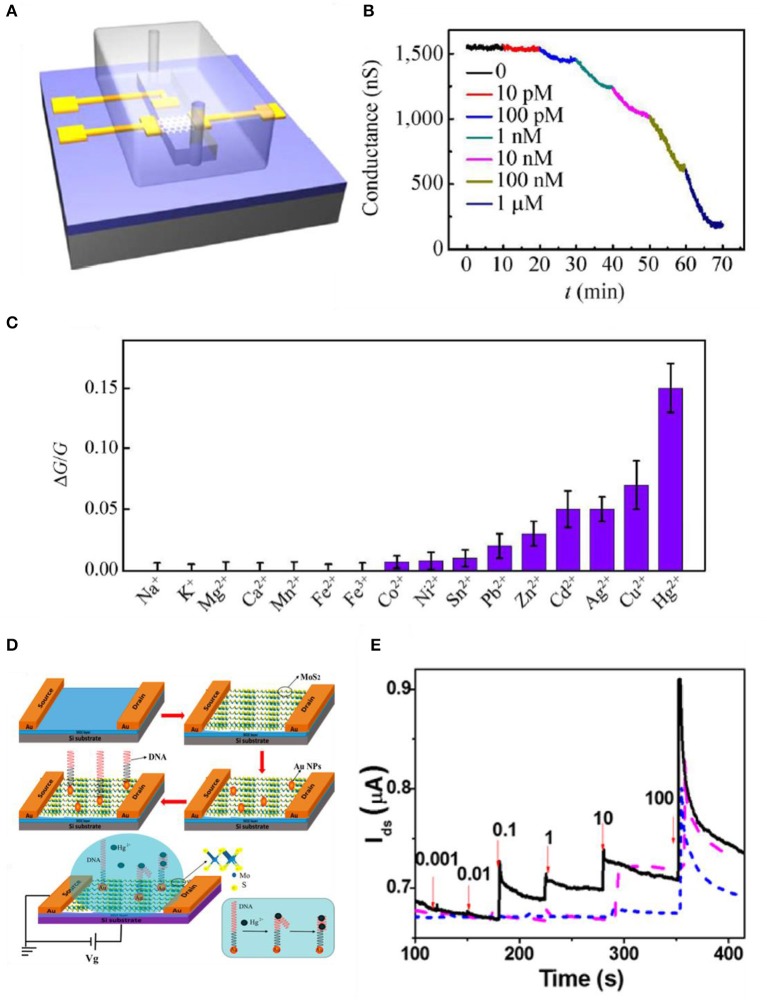
MoS_2_-based FET metal ion sensors. **(A)** Schematic diagram of microfluidic channel onto MoS_2_ device. **(B)** Sensing curve data using low-concentration Hg^2+^ ions. **(C)** Selectivity to various metal ions having a concentration of 1 nM. Figures **(A–C)** were reproduced from Jiang et al. (2015) with permission from Springer. **(D)** Illustration of manufacturing process of MoS_2_ based FET. MoS2/DNA-Au NPs hybrid structure is shown. **(E)** Real-time sensing curves of Hg^2+^ (nM) in water (*V*_DS_ = 0.1 V), MoS_2_/DNA-Au NPs (black, solid), MoS_2_-Au NPs (purple, dash), MoS_2_ (blue, short dash). Figures **(D,E)** were reproduced from Zhou et al. (2016) with permission from the American Chemical Society.

TMDs also have high binding energy to heavy metal ions due to their chalcogenide ions. Jiang et al. used MoS_2_-based FET to sense mercury ions. MoS_2_ has a high affinity to Hg^2+^ due to their sulfur sites that are bonded extremely strong (Jiang et al., 2015). Binding with mercury ions leads to a p-type doping effect and eventually reduces the electron concentration. This affects the electrical and optical features of MoS_2_ such as electron transport and photoluminescence properties. The sensitivity of the device is confirmed by monitoring the conductance change when mercury ions are introduced. As shown in Figure 8A, flakes of MoS_2_ were mechanically exfoliated to a Si/SiO_2_ substrate, and then electron-beam lithography and electron-beam evaporation were used to define contact electrodes. After the process, a polydimethylsiloxane (PDMS) microfluidic channel was introduced to the device for Hg^2+^ solution delivery. The Hg^2+^ solution was made by dissolving Hg(ClO_4_)_2_ in DI water. In Figure 8B, to check the sensing properties of the device, Hg^2+^ solutions possessing various concentrations flowed into the device. As the concentration increased, the conductance declined due to charge impurity-induced scattering effects. As Hg^2+^ increases, the p-type doping effect also increases (transfer curves shift to the right as a higher concentration of Hg^2+^ is introduced), leading to electrons to decrease, resulting in the reduction of carrier mobility. Also, in Figure 8C, selectivity was confirmed by testing with various heavy metal ions whose concentrations were all 1 nM. Zhou et al. also detected Hg^2+^ with MoS_2_ by decorating specific DNA functional groups. Figure 8D indicates the fabrication process of the MoS_2_/DNA-Au nanoparticle hybrid structure. MoS_2_ nanosheets were formed by Li ion exfoliation at RT. MoS_2_ powder was intercalated by lithium ions and then they were sonicated in water. MoS_2_ crystals were formed and then immersed into a butyllithium solution for a week filled with Ar gas. Li_x_MoS_2_ was retrieved by centrifugation and was washed to eliminate the residues. Ultrasonication was processed for exfoliation to form nanosheets. Then, the film was transferred onto a SiO_2_ substrate as shown in Figure 8D. Au nanoparticles were deposited on the film by sputtering, resulting in 2-nm decoration on the surface. Then, the DNA solution was injected on the active site of the device and was rinsed with deionized water to eliminate redundant DNA. When Hg^2+^ is introduced to the device, T-(Hg^2+^)-T chelates, formed by the reactions between Hg^2+^ and the thymidine of the DNA molecules resulting in electrical properties to change MoS_2_. Real-time detections of Hg^2+^ in water are illustrated in Figure 8E. The black curve indicates the platform of MoS_2_/DNA-Au nanoparticles, the purple dash curve indicates MoS_2_-Au nanoparticles, and the blue dash curves indicate MoS_2_ only. From the curves in Figure 8E, it is noticed that the MoS_2_ has higher conductivity as Hg^2+^ concentration increases. The results are opposite from Jiang et al., which explains that MoS_2_ from Zhou et al. is a p-type semiconductor. From the fabricating process, MoS_2_ was exposed to an oxygen-containing atmosphere and the oxygen molecules were adsorbed onto the defects or sulfur sites on the layer of MoS_2_ film and eventually worked as p-type dopants by trapping electrons (Zhou et al., 2016).

Li et al. fabricated thin-layered black phosphorus (BP) Hg^2+^ sensors by mechanically exfoliating BP with adhesive tape and transferring it to the Si/SiO_2_ substrate. The source, drain, and the gate was defined by photolithography and then sputtered with Cr and Au. The device was introduced to 0.1 M sodium acetate buffer solution with pH = 4.6 with various Hg^2+^ concentrations from 0.01 to 100 ppb at RT. The concentration increases of Hg^2+^ led to the transfer curves to reduce in current, suggesting an increase of resistivity of the BP channel. BP is a typical p-type semiconductor, which means holes are the main charge carriers. When Hg^2+^ ions are attached to the surface of BP, they act as a positive gate voltage. In other words, Hg^2+^ ions repel positively charged holes. Consequently, the Fermi energy shifts from the valence band (i.e., charge neutrality point shifts to the negative direction) and hole density reduces, resulting in BP conductivity decline (Li et al., 2017).

## Other Chemical Sensing

### Dopamine, Ascorbic Acid, and Uric Acid Sensing With Various 2D Materials

**Figure 9:**
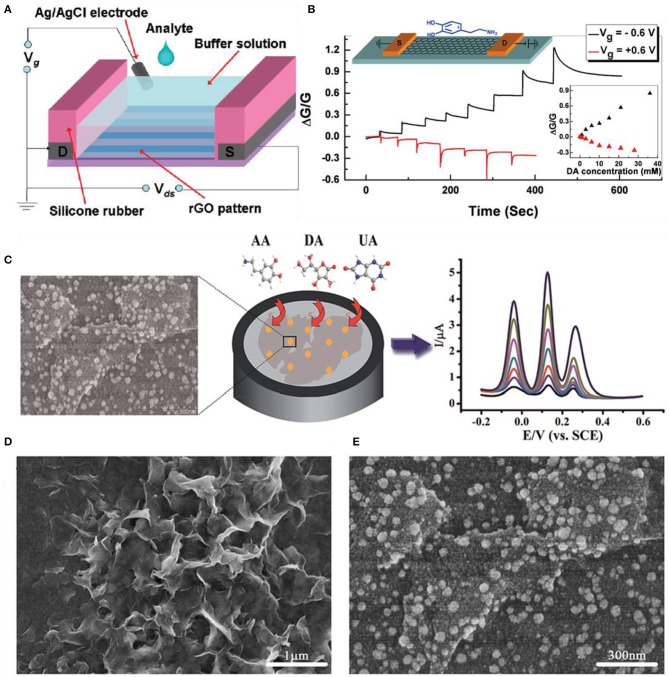
Dopamine, uric acid, and ascorbic acid sensors using 2D materials. **(A)** Schematic figure of RGO FET for dopamine sensing. **(B)** Detection curves of RGO FET at *V*_g_ = 0.6 V; each step indicates the gradual addition of dopamine ranging from 1 to 8 mM. Figures **(A,B)** were reproduced from He et al. (2010) with permission from the American Chemical Society. **(C)** Schematic illustrations of gold-decorated MoS_2_-based electrochemical sensors. **(D)** Scanning electron microscopy images of pristine MoS_2_ and **(E)** gold nanoparticle-decorated MoS_2_. Figures **(C–E)** were reproduced from Sun et al. (2014) with permission from the Royal Society of Chemistry.

Dopamine (DA), ascorbic acid (AA), and uric acid (UA) are very essential to human metabolism because they influence the central nervous system and serum by coexisting. The lack or excess of DA, AA, and UA may lead to various disorders that can be fatal to life. The importance of direct, simultaneous, quick sensing of DA, AA, and UA cannot be emphasized. Overall, 2D material-based DA, AA, and UA sensors accompany doping, metal nanoparticle decoration, and reduction process to enhance selectivity and sensitivity. He et al. manufactured a dopamine device based on RGO FET by reducing patterned GO films. Figure 9A shows the schematic diagram of the experimental setup of the top-gate (solution gated) RGO FET for dopamine sensing. After patterning GO with the capillary method, the chemical reduction process was followed by hydrazine vapor. Before micropatterning, the SiO_2_ substrate was modified with 3-aminopropyltriethoxysilane (APTES) to supply a positively charged surface. This process provides the uniformity of GO patterns and prevents aggregation on the substrate. Figure 9B shows the detection of dopamine in the RGO FET device at gate voltages *V*_g_ = 0.6 V, −0.6 V. Each step indicates the addition of dopamine increasing the concentration ranging from 1 to 8 mM. Dopamine can easily bind with RGO through strong π-π interactions. Also, the transfer curves shifted to the right, showing that they induce p-doping or incline the hole concentration in the RGO film (He et al., 2010). Yang et al. demonstrated a typical RGO sensor for simultaneous detection of dopamine, ascorbic acid, and uric acid. The GO sheet was synthesized by the Hummers method and they were electrochemically reduced. The experiments were done with cyclic voltammograms. Each peak current is proportional to the concentration of each solution. The detection limits for DA, UA, and AA are 0.5, 0.5, and 250 μM, respectively. Moreover, not only measuring with DA, UA, and AA but also mixing with other inorganic ions such as 100-fold K^+^, Ca^2+^, Na^+^, Mg^2+^, Zn^2+^, ${\text{NH}}_{4}^{+}$, Cl^−^, ${\text{SO}}_{4}^{2-}$, ${\text{NO}}_{3}^{-}$, and ${\text{HCO}}_{3}^{-}$ do not interfere with the main peaks, indicating that the sensor has great selectivity toward DA, UA, and AA (Yang et al., 2014). Other groups used graphene doping Pt to form nanocomposites (Sun et al., 2011) or doping nitrogen (Sheng et al., 2012). Yang et al. formed graphene/Pt nanoparticle composites by dissolving PtCl_4_ in ethylene glycol containing 0.1 M NaOH. After vigorously stirring, graphene powders were added to the Pt colloidal solution and then the solution was ultrasonicated. After filtration and drying process, the Pt/graphene catalyst was obtained. The experiments were done using cyclic voltammograms comparing bare glassy carbon (GC), graphene, and graphene/Pt nanocomposite electrodes. Anodic peak currents were 102 μA (GC), 183 μA (graphene), and 371 μA (graphene/Pt). From these results, both graphene and graphene/Pt-modified electrodes increase the electrochemical sensitivity. The amperometric responses of AA, DA, and UA were measured. All experiments were progressed in a 0.1 M phosphate buffer solution at 0.5 V and the detection limits were 0.15 μM (AA), 0.03 μM (DA), and 0.05 μM (UA). Sheng et al. doped nitrogen on graphene for the simultaneous determination of AA, DA, and UA. After mixing GO and melamine, the mixture was annealed to 800°C for 1 h in Ar atmosphere. Then, the final product was taken out from the tube. The electrochemistry of each solution was measured. All three cases had excellent electrocatalytic activity toward the oxidation of AA, DA, and UA. The interactions between the target biomolecules (hydrogen bonds), nitrogen-doped graphene (π-π stack), structure of the molecules, and the unique structural and properties of nitrogen-doped graphene influenced the electrocatalytic activity.

Sun et al. decorated Au nanoparticles onto MoS_2_ nanosheets for the detection of AA, DA, and UA. In Figure 9C, the overall characterization, fabrication, and electrochemical sensing process are illustrated. The MoS_2_ nanosheets were prepared by the intercalation exfoliation method. The Au nanoparticles were decorated by the electrochemical deposition method. The synergetic effects of MoS_2_ and Au nanoparticles enhanced the oxidation reactions of AA, DA, and UA. Figures 9D,E are scanning electron microscopy images of pristine MoS_2_ and Au nanoparticle-decorated MoS_2_ nanostructures on ITO. The detection limits for AA, DA, and UA were 100, 0.05, and 10 μM, respectively. Wu et al. electrochemically reduced MoS_2_ single-layered nanosheets to detect various biomolecules such as DA in the presence of AA and UA. The oxidation peak of AA was much lower than that of DA or UA. The DA oxidation signal being stronger than AA or UA is due to the negative charges existing on reduced MoS_2_, which repel the anionic AA and UA but attract the cationic DA. The peak current of DA increased linearly with the DA concentration from 1 to 50 μM (Wu et al., 2012).

### Nucleic Acid Sensing With Various 2D Materials

**Figure 10:**
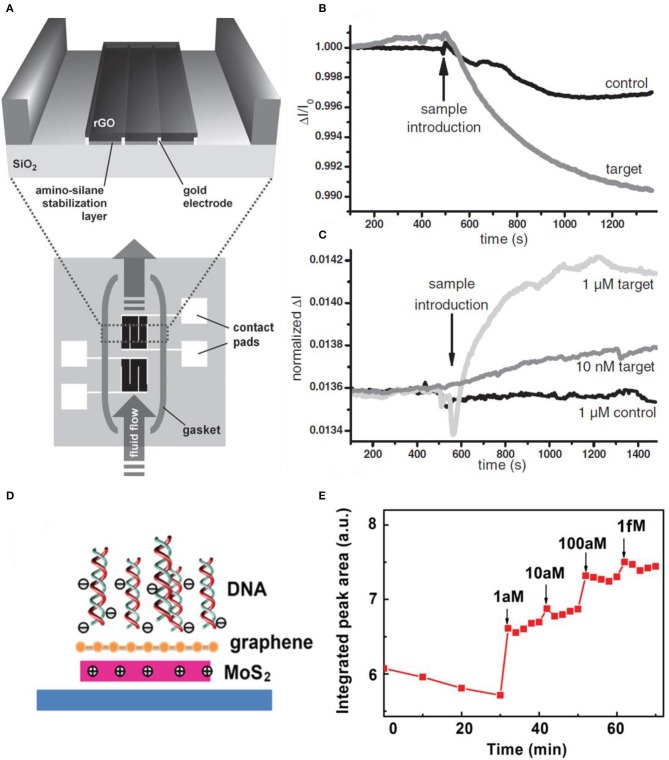
RGO FET for real-time DNA detection. **(A)** Schematic diagram of RGO FET. RGO is deposited on top of the pre-fabricated electrodes. The two devices are isolated, to eliminate non-specific biological adhesion (bottom), which acts as a reference device. **(B)** RGO FET sensing curves toward DNA. Non-specific binding (control curve) occurs to the first device (bottom device in **(A)**. **(C)** Second device curves having much more selectivity toward the target DNA. Figures **(A–C)** were reproduced from Stine et al. (2010) with permission from Wiley. Graphene/MoS_2_ heterostructures for detection of DNA. **(D)** Scheme of the charge distribution when DNA becomes adjacent. **(E)** Photoluminescence response of graphene/MoS_2_ to increased concentration of the target DNA. Figures **(D,E)** were reproduced from Loan et al. (2014) with permission from Wiley.

Biomolecule detecting and identifying in a solution is gaining importance these days due to the use of various areas such as medical diagnostics, food analysis, and state-of-the-art technology and science. Existing techniques of biomolecule sensing were based on fluorophores or enzymes, which have great sensitivity but cannot be monitored in real time; in other words, the response takes a long time. Also, the cost and complexity of monitoring for biological agents are high. To reduce the complexity of traditional biomolecule sensors, many types of researches and studies were based on detecting the target molecules by binding directly to the sensor. Surface plasmon resonance (SPR) was emerging for label-free biological detection (Homola, 2008). Recently, biomolecule sensors based on silicon nanowire (Curreli et al., 2008; Stern et al., 2008) and carbon nanotube (Allen et al., 2007) FETs have higher sensitivities. These enhanced nanoscale sensors are due to their high surface area-to-volume ratios; thus, only a small number of charged molecules are needed to change the electrical characteristics of the FET (Cui et al., 2001). These nanowire/tube devices have difficulties with alignment and reproducibility issues. Recently, using 2D materials such as graphene, GO, RGO, and TMDs is emerging due to easy fabrication (exfoliation process, functionalization), low cost, and high sensitivity. In particular, GO has oxygen group functions and defects that bond selectively with biomolecules and they are hydrophilic. RGO also has functional groups and high conductivity. Stine et al. deposited RGO onto the SiO_2_ substrate and detected DNA in real time. Figure 10A shows the schematic diagram of the RGO FET. The device is fabricated into two devices, which are isolated inside the gasket. The bottom device acts as a reference to eliminate non-specific DNA adhesion. The magnified area is the deposition of GO on top of the pre-fabricated electrodes. The GO is reduced by hydrazine exposure. Figure 10B shows the single FET device having no specificity to the target DNA. The target DNA (complementary DNA) has more conductance difference than the controlled specimen (non-complementary DNA) but the sensor clearly responded to both cases. Using the second FET solved the selectivity problem as plotted in Figure 10C. One device was activated as an internal reference, and there was no response to the control sample. The existence of reference led to selectivity toward the target DNA, and also non-complex functionalization was needed (Stine et al., 2010). Yin et al. demonstrated decorating Pt nanoparticles onto a conductive RGO thin film by photochemical reduction. The DNA detection was based on the Pt-S covalent bonding and immobilizing the DNA. The transfer curves shifted to the negative direction, meaning the DNA caused n-doping effects. Due to the selective effect of Pt nanoparticles, the shift of Pt nanoparticles/RGO was much larger than pristine RGO. The DNA detection limit of real-time measurements was 2.4 nM (Yin et al., 2012).

Loan et al. designed a heterostructural structure by stacking graphene on MoS_2_, providing ultrasensitivity to DNA. The role of graphene is to protect the reaction between MoS_2_ and the surrounding environment and to be a biocompatible superficial layer to immobilize DNA molecules on its surface. The photoluminescence intensity of the MoS_2_ from the stack of graphene/MoS_2_ increased as the concentration of DNA increased by adding them. Real-time measurements were available in a few minutes and the detection limit was reached to the level of aM. In Figure 10D, the schematic of the heterostructure is depicted. When DNA approaches the heterostructure, the DNA adsorption on the composite results in more positive charges in the MoS_2_, which enhances its PL intensity. Also, graphene is not affected by the DNA sensing by confirming the Raman spectra, suggesting that the Fermi energy of graphene is not changed by DNA adhering. This is due to the electron transfer of MoS_2_ to graphene, which means any induced hole of graphene is filled by the electron from MoS_2_, resulting in MoS_2_ becoming reduced due to the loss of electrons. Figure 10E is the plot of PL experiments having greater intensity as the concentration of DNA is increased (Loan et al., 2014).

### Amino Acid and Protein Sensing With Various 2D Materials

**Figure 11:**
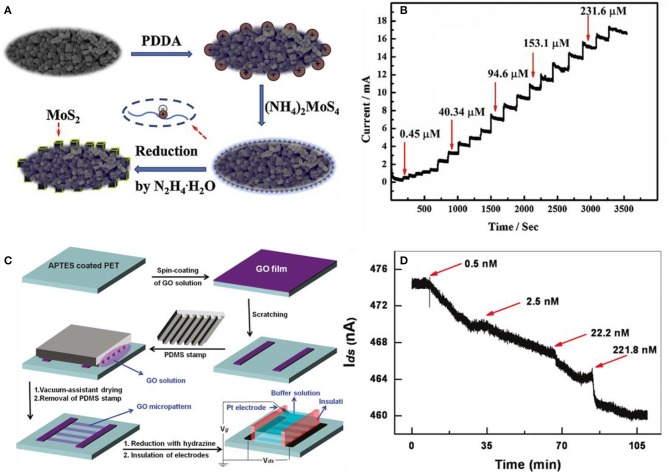
p-type MoS_2_ nanocube modified poly(diallyl dimethylammonium chloride)–mesoporous carbon composite for the L-cysteine sensor. **(A)** Schematic illustration of depositing MoS_2_ nanoparticles on mesoporic carbon (MC). After coating poly(diallyl dimethylammonium chloride) on MC, MoS_2_ is decorated by reducing ${\text{MoS}}_{4}^{-}$. **(B)** Response curves to various concentrations of L-cys at the MoS_2_/PDDA-MC/GCE composite in 0.1 M phosphate buffer solution. Figures **(A,B)** were reproduced from Zheng et al. (2015) with permission from Elsevier. Pyrene functionalized RGO-based thin-film transistors (TFT) for detection of fibronectin. **(C)** Fabrication process of RGO based TFT. **(D)** Fibronectin detection by the increase of concentration at the pyrene-functionalized RGO TFT sensor. Figures **(C,D)** were reproduced from He et al. (2011) with permission from the American Chemical Society.

Protein is the basic unit that makes up any living organism. Protein and amino acid detection are crucial in medical diagnosis (Yoo et al., 2014), food security (Setford et al., 2002), and biotechnology (Zhang et al., 2016). These molecules are also compatible with 2D materials as nucleic acids. However, amino acid and protein are much larger in molecular weight and size than nucleic acids. L-cysteine (L-Cys) is an example of a crucial amino acid, which plays a vital role biologically. This amino acid can be used as an indicator for several diseases (Shahrokhian, 2001), and L-Cys has been applied in industrial purposes such as cosmetics, food procedure, and pharmacy uses (Lima et al., 2008). Zheng et al. designed MoS_2_-modified poly(diallyl dimethyl ammonium chloride) (PDDA)–mesoporous carbon (MC) composites to detect L-Cys. The fabrication process is illustrated in Figure 11A. Functionalizing MC with PDDA was the first step and then MoS_2_ nanocubes were decorated onto PDDA-MC by the reduction process of (NH_4_)_2_MoS_4_ using the hydrothermal method. L-Cys was demonstrated with amperometric responses plotted in Figure 11B having increased current steps by the elevated concentration of L-Cys (Zheng et al., 2015). He et al. fabricated transparent, flexible RGO thin-film transistors (RGO TFT) to detect fibronectin, which is a type of protein. In Figure 11C, the RGO-based thin-film transistor fabrication is illustrated. GO was first spin-coated on a 3-aminopropyltriethoxysilane (APTES)-modified PET substrate. The APTES helps GO to be adsorbed to the substrate and prevents aggregation. Then, GO was reduced with hydrazine vapor to obtain RGO films. For selectivity, the RGO channel was functionalized with 1-pyrenebutanoic acid succinimidyl ester, which acted as a linker molecule to seize the proteins in the buffer solution. As shown in Figure 11D, the current of the functionalized RGO channel decreased as the fibronectin was added. This is due to the n-doping effect of fibronectin. Also, He et al. tested the detection of avidin with other functional groups. The RGO channel was coated with polyethyleneimine (PEI) and polyethylene glycol (PEG) and then biotinylated using biotin-N-hydroxysuccinimide ester (He et al., 2011). Mukherjee et al. used a liquid-gated FET graphene decorating aptamer with the linker of the pyrene group to detect adenosine triphosphate (ATP) (Mukherjee et al., 2015). Ohno et al. also designed aptamer-modified graphene to detect immunoglobulin E (IgE), and other proteins such as bovine serum albumin (BSA) and streptavidin (SA) had no response to the device (Ohno et al., 2010).

## Conclusion and Perspective

**Figure 12:**
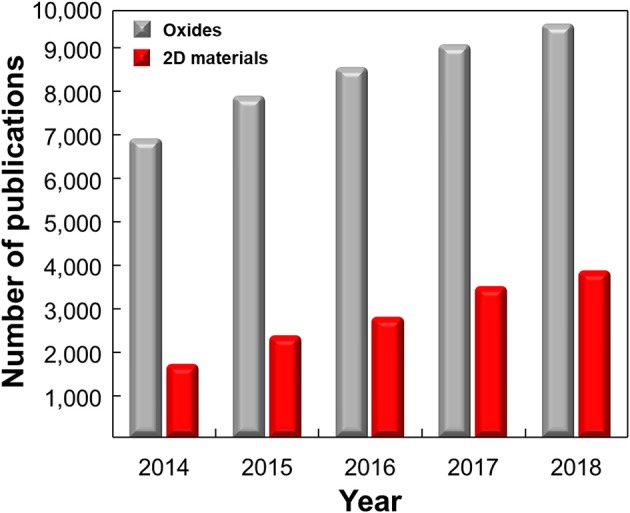
Number of publications on ion/molecule sensors based on 2D materials and metal oxides by years. The data were collected using the Web of Science [v. 5. 32]–Web of Science Core Collection Search: keywords of ((“hydrogen ion” OR glucose OR Pd^2+^ OR Sr^2+^ OR Cu^2+^ OR Cd^2+^ OR Hg^2+^ OR Pb^2+^ OR Cr^4+^ OR Ni^2+^ OR Co^2+^ OR Mn^2+^ OR uric OR ascorbic OR dopamine OR DNA OR amino OR protein sens*) AND (graphene OR MoS_2_ OR molybdenum disulfide OR black phosphorous OR MoSe_2_ OR molybdenum selenide OR WS_2_ OR tungsten disulfide OR WSe_2_ OR tungsten diselenide)) and ((“hydrogen ion” OR glucose OR Pd^2+^ OR Sr^2+^ OR Cu^2+^ OR Cd^2+^ OR Hg^2+^ OR Pb^2+^ OR Cr^4+^ OR Ni^2+^ OR Co^2+^ OR Mn^2+^ OR uric OR ascorbic OR dopamine OR DNA OR amino OR protein sens*) AND (oxide)) were used.

The promising physical and chemical properties of 2D materials have encouraged lots of studies on developing highly selective ion/molecule sensors utilizing surface defects, large specific surface area, and easy surface functionalization. Many researchers are seeking to use 2D materials as ion/molecule sensors for various reasons such as environmental monitoring, real-life healthcare, and food intake applications. Although studies on 2D material-based ion/molecule sensors are in the early stage compared to oxide-based sensors (Figure 12), various approaches to achieve high selectivity using 2D materials summarized in this review will benefit when appropriate sensor platform for IoT applications is developed and will result in the realization of e-tongue or taste sensors. To fulfill the advent of taste sensors or e-tongue, the exact one-to-one correspondence of the sensor and target material should be available and the device should discriminate the composition of substance precisely. Despite these efforts, they are still far behind commercialization and will still face lots of obstacles toward usage in the real world. The highly selective receptors with high reliability should be discovered, and reproducible sensor fabrication with a uniform sensor performance should be secured for mass production. In particular, targeting substances in liquid media causes difficulties in developing suitable sensor structures for mobile application and further studies are necessary.

## Author Contributions

The manuscript was written through contributions of all authors. All authors have given approval to the final version of the manuscript.

### Conflict of Interest

The authors declare that the research was conducted in the absence of any commercial or financial relationships that could be construed as a potential conflict of interest.
